# Hume on the Prospects for a Scientific Psychology

**DOI:** 10.1002/jhbs.70050

**Published:** 2026-04-16

**Authors:** Michael Jacovides

**Affiliations:** ^1^ Department of Philosophy Purdue University West Lafayette Indiana USA

**Keywords:** Associationism, Faculty Psychology, Hume, Newton, Scientific Psychology

## Abstract

In Section One of an *Enquiry Concerning Human Understanding*, Hume distinguishes between two sorts of writing on human nature: first, one that appeals to common sense to make virtue seem attractive and, second, one that attempts to describe the principles governing the mind. Hume's defence of the second approach is in part a defence of the possibility of scientific psychology. Within the second approach, he distinguishes two parts: first, a descriptive branch he calls ‘mental geography’ and, second, a branch he compares to Newton's project in astronomy. In his defence of mental geography, Hume sketches an account of his method of enquiry in psychology. Common sense describes some basic faculties, philosophers can make finer distinctions within these, and introspection allows us to reliably describe ground‐level processes. Hume's vision of Newtonian psychology is one that appeals to laws and forces and finds the hidden springs of the mind. His attempt to explain causal inference by appealing to the transfer of vivacity across associated perceptions in Part 2 of Section 5 is an attempt at Newtonian psychology: it's speculative, explanatory, and enunciates a putative psychological law.

## 1 Easy and Abstruse Psychology

### 1.1 Two Species of What?

In Section 1 of his *Enquiry Concerning Human Understanding*, Hume draws a distinction between two approaches, one easy and one abstruse, to what he calls “moral philosophy, or the science of human nature” (*EHU* 1.1).^1^ The goal of those taking the first approach is to paint virtue “in the most amiable colours; borrowing all helps from poetry and eloquence, and treating their subject in an easy and obvious manner” (*EHU* 1.1). The goal of those taking the second approach is “to find those principles, which regulate our understanding, excite our sentiments, and make us approve or blame any particular object, action, or behaviour” (*EHU* 1.2). That is, the goal of the second approach is to find the principles that govern the mind. He argues that the second approach can reach the level of science, which is to say he wants to defend the prospects of a scientific psychology. In his description of a scientific psychology, he isn't merely considering a possible future of a possible science of mind. He is also advertising and framing the achievements of his work.

We may call the generic discipline that Hume is interested in in the opening lines of the *Enquiry* ‘psychology.’ The first approach is interested in the applications of psychology to life to help us live better and more sociable lives. The second is interested in discovering the principles regulating the understanding, passions, and our moral affections.

The first uses of the word ‘psychology’ are from the 16th century to describe the science of the soul.^2^ One of the first uses of the English term to describe the science of the mind without making it the study of a substantial soul is by David Hartley, in his *Observations on Man*, a work published the year after Hume's *Enquiry Concerning Human Understanding*.^3^ Hartley divides natural philosophy into the following parts: “Mechanics, Hydrostatics, Pneumatics, Optics, Astronomy, Chemistry, the Theories of the several manual Arts and Trades, Medicine and Psychology, or the Theory of the human Mind, with that of the intellectual Principles of Brute Animals” (Hartley 1749, 1.354).

In the first chapter of his book, Hartley says that he develops his doctrine “from what Mr. Locke and other ingenious persons since his Time, have delivered concerning the Influence of *Association* over our Opinions and Affections” (1749, 1.5). Barbera Oberg reasonably remarks, “Given Hartley's wide reading in theology and philosophy—Locke, Joseph Butler, Descartes, Malebranche, Leibniz—and his acknowledgement that he had read other sources on the subject of association, it is probable that he would also have read Hume” (1976, p. 441).

Oberg also asserts, “for Hobbes, for Locke, and for Hume… the theory of association was only incidental to other concerns. For Hartley, the association of ideas explained the working of the human mind” (1976, p. 453). This contrast, however, is incompatible with Hume's own presentation of his work. In the abstract of the *Treatise*, written under a guise of anonymity, he claims, “Thro’ this whole book, there are great pretensions to new discoveries in philosophy; but if any thing can intitle the author to so glorious a name as that of an *inventor*,'tis the use he makes of the principle of the association of ideas, which enters into most of his philosophy” (Abstract 2007, ¶35). Given that Hume very probably influences Hartley's account and given the centrality of principles of association to both of their projects, it would be unreasonable to say that Hartley is doing psychology while Hume is doing something else.

The terminology Hume uses implies that he thinks that the successful pursuit of abstruse psychology would constitute a science. The second approach to the science of man aims at finding “those original principles, by which, in every science, all human curiosity must be bounded” (*EHU* 1999, 1.2). In defence of this approach, Hume writes, “The sweetest and most inoffensive path of life leads through the avenues of science and learning” (*EHU* 1.10). As Hume understands it, the second approach to psychology has two parts, one of which attempts “barely to know the different operations of the mind” and constitutes, “no inconsiderable part of science” (*EHU* 1.13). He reassures us about the possibility of this kind of mental description by saying that we shouldn't worry “that this science is uncertain and chimerical” (*EHU* 1.14). In describing his hopes for a second, explanatory description of the laws governing the mind, Hume says that others have pursued this project with partial success and that “more ardent application may bring these sciences still nearer their perfection” (*EHU* 1.15).

One might reasonably wonder whether the terms in Hume's initial disjunctive description of the general topic (‘moral philosophy’ or ‘the science of human nature’) refer to the same discipline. Moral philosophy seems like an odd expression to describe the science that describes the principles that regulate human understanding. ‘Professor of Ethics and Pneumatical Philosophy,’ the academic position that Hume doesn't receive in 1745 at the University of Edinburgh, shows that there's a cultural and institutional impulse there to combine the study of mind with the study of morals (Stewart 2002, p. 86). Sometimes, as in the subtitle of the *Treatise of Human Nature* and in his comparison of moral and mathematical sciences at the beginning of Section 7 of the first *Enquiry*, Hume uses the term ‘moral’ as broadly he does here, so as to include all the principles that regulate the human mind. More often he uses ‘morals’ as we do to mean *ethics*, for example in the discussion elsewhere in the first section about the foundations of morals (*EHU* 1.2) and in title of the second *Enquiry* (“Concerning the Principles of Morals”). The first line of Francis Hutcheson's *System of Moral Philosophy*, helps us make sense of Hume's usage: “The intention of moral philosophy is to direct men to that course of action which tends most effectually to promote their greatest happiness and perfection; as far as it can be done by observations and conclusions discoverable from the constitution of nature” (1755, 1.1). The core of moral philosophy is to teach us how to live, but in the periphery lie empirical questions of human nature that will help us answer that question.

The terms that Hume uses for the first approach to this sort of enquiry, namely, ‘easy,’ ‘obvious,’ and ‘humane,’ all seem apt given the other things that he says about such approaches. None of the terms that he gives for the second approach seem entirely well chosen, either because they don't fit the way that this sort of study of the mind is actually practiced by his predecessors or because they don't fit the way that Hume thinks that they ought to be practiced. He thinks that, as actually practiced, most attempts at the second sort of enquiry end up on the wrong track; so, I say, it's misleading to call them ‘accurate.’ If they are ‘profound,’ it's only in their ambition. On the other hand, he thinks that the first principles of psychology ought to be discovered by experience, so it's a little misleading to call the science he has in mind ‘abstract’. He calls the second approach to the science of man ‘abstruse,’ which means difficult, but in the last paragraph of the section, he expresses the hope that “reasoning in this easy manner, we can undermine the foundations of an abstruse philosophy, which seems to have hitherto served only as a shelter to superstition” (*EHU* 1.17). He would like to write a work in an easy style that persuades his reader to abandon the difficult and obscure philosophy that's been used to justify religious error.

### 1.2 The Challenge of Common‐Sense Psychology

According to Hume, easy psychology is more reliable than previous attempts at abstruse psychology. A humane psychologist isn't trying to come up with original hypotheses about the workings of the mind. In the project of inspiring good thoughts and good behaviour, he assumes the truth of common‐sense psychology and is thus less likely to persist in error: “if by accident he falls into error, goes no farther; but renewing his appeal to common sense, and the natural sentiments of the mind, returns into the right path, and secures himself from any dangerous illusions” (*EHU* 1.4).

In contrast, abstruse philosophers are indifferent towards what most people think. They set off from premises that seem true to them and embark on a chain of inferences. When there's one mistake in the chain, the whole endeavour goes off the rails: “one mistake is the necessary parent of another, while he pushes on his consequences, and is not deterred from embracing any conclusion, by its unusual appearance, or its contradiction to popular opinion” (*EHU* 1.4). The practitioner of this sort of enquiry into the mind gains a temporary reputation “from the caprice or ignorance of their own age,” but such philosophers haven't acquired a reputation from later thinkers, who are better judges (*EHU* 1.4).

We might doubt Hume's sincerity in his endorsement of the reliability of common sense. In his essay “The Standard of Taste,” he implies that there is a tension between common sense and the sceptical philosophy, though in the particular case under discussion, the subjectivity of aesthetic taste, common sense is said to agree with sceptical philosophy (“common sense, which is so often at variance with philosophy, especially with the sceptical kind, is found, in one instance at least, to agree in pronouncing the same decision,” “Standard,” 1985, 226). In spite of this purported tension, every time Hume says in his own voice that some doctrine represents common sense, he endorses the doctrine. Common sense tells us, he tells us, that the desire for reward causes action (*T* 2.3.2.5), that revolt against tyrants is morally permissible (*T* 3.2.9.4), and that perfect equality of possessions is impractical (*EPM* 1999, 3.26, see also *T* 1.3.13.4, 2.3.8.8, 3.2.10.7, *EPM* 1.2). His preferred version of scepticism, mitigated or academic scepticism, is the result of correcting Pyrrhonism “by common sense and reflection” (*EHU* 12.24).

The lesson of these passages is that Hume thinks that common sense is generally reliable, and that the challenge of doing better than common sense is a weighty one. Common‐sense psychology can call upon the collective experience of humanity and on our innate inclinations. A scientific psychology that purports to be superior to common sense and also independent of common sense has its work cut out for it.

The core topics of the abstruse investigation that Hume sketches in the first section of the first *Enquiry* are the principles that regulate the mind. He envisions that this discipline may surpass common sense and that it is or may become a science. He has a vision of a scientific psychology, and we should be curious to learn what it is.

## 2 Mental Geography

### 2.1 The Possibility of Mental Geography

Hume defends the possibility of scientific psychology by dividing the prospective field in two: a descriptive part and an explanatory part. The first he calls “mental geography”; we might call the second Newtonian psychology. For both fields, he gives general reasons for believing in their possibility, and he also presents paradigms of success as models for emulation and reasons for hope.

By “mental geography,” Hume means the “delineation of the distinct parts and powers of the mind” (*EHU* 1.13). Its goal is “barely to know the different operations of the mind, to separate them from each other, to class them under their proper heads” *(EHU* 1.13). Mental geography, as Hume uses the expression, is merely descriptive and taxonomic. Once we have built a solid and certain description of mental phenomena, he hopes to find explanatory laws and forces that explain those phenomena.

Hume concedes that it's difficult to describe mental activity. “The operations of the mind” are “most intimately present to us,” when we reflect on them; even so, he tells us, they seem “involved in obscurity” such that we can't “readily find those lines and boundaries, which discriminate and distinguish them” (*EHU* 1.13). Mental objects are delicate, so they change; they are “too fine to remain long in the same aspect or situation; and must be apprehended in an instant,” so to understand them, one needs “superior penetration, derived from nature, and improved by habit and reflection” (*EHU* 1.13).

In Section 1 of the first *Enquiry*, Hume gives two considerations in defence of the possibility of mental geography. The first appeals to the successes of his contemporaries. In the first two editions, he cites Francis Hutcheson and Joseph Butler as thinkers who have successfully carried out mental geography. Hutcheson showed that the faculty by which we distinguish truth from falsehood is distinct from the faculty by which we distinguish virtue and vice “by the most convincing arguments” (*EHU* p. 232). Butler showed the impropriety of the standard division between selfish and benevolent passions and “prov'd, beyond all Controversy, that even the Passions, commonly esteem'd selfish, carry the Mind beyond Self, directly to the Object” (*EHU* p. 234). “These two instances,” according to Hume, “may suffice to show us the Nature and Importance of this Species of Philosophy” (*EHU* p. 234). Hume thinks that since Butler and Hutcheson succeeded, so can we. How much this justifies mental geography depends on one's opinion of Butler's and Hutcheson's work.

In later editions of the *Enquiry*, Hume takes the details out of his successful case studies and gives a truncated justification: “Some instances, especially late ones, of success in these enquiries, may give us a juster notion of the certainty and solidity of this branch of learning” (*EHU* 1.14). Hume thinks of himself as being in a tradition with Butler and Hutcheson, and he thinks that their successes bode well for his project. In a similar argument in the *Treatise*, Hume places himself in a tradition including not just Butler and Hutcheson, but also Locke, Shaftesbury, and Mandeville (*T* 2007, Intro, p 7).

### 2.2 Divide and Describe

The second reason Hume gives for the possibility of mental geography is best understood as a justificatory description of the method he'll use in the *Enquiry*. This justification has two parts. The first appeals to our capacity for reflection. According to Hume,

It cannot be doubted, that the mind is endowed with several powers and faculties, that these powers are distinct from each other, that what is really distinct to the immediate perception may be distinguished by reflection; and consequently, that there is a truth and falsehood in all propositions on this subject, and a truth and falsehood, which lie not beyond the compass of human understanding.

(*EHU* 1.14)

The straightforward way to make sense of this argument is to suppose that the objects of “immediate perception” include faculties. Otherwise, it seems as if the parts of the argument won't fit together.

On the other hand, the faculties that Hume says in Section 1 of the *Enquiry* are known to everyone are all present in the *Treatise*, but don't seem to be immediately epistemically accessible. There he famously declares, “when I enter most intimately into what I call *myself*, I always stumble on some particular perception or other, of heat or cold, light or shade, love or hatred, pain or pleasure. I never can catch *myself* at any time without a perception, and never can observe any thing but the perception” (*T* 2007, 1.4.6.3), which seems to exclude knowledge of our faculties by reflection. Indeed, the theory of the mind offered at the end of that chapter on personal identity seems to restrict the constituents of the mind to perceptions: “the true idea of the human mind, is to consider it as a system of different perceptions or different existences, which are link'd together by the relation of cause and effect, and mutually produce, destroy, influence, and modify each other” (*T* 1.4.6.19). We might follow Tamás Demeter (2021 5372) in concluding that Hume doesn't think faculties are accessible to introspection.

If that's right, however, how is the distinguishability of perceptions supposed to help us with our descriptions of faculties? Hume appeals to the distinctions drawn by the common folk:

There are many obvious distinctions of this kind, such as those between the will and understanding, the imagination and passions, which fall within the comprehension of every human creature; and the finer and more philosophical distinctions are no less real and certain, though more difficult to be comprehended.

(*EHU* 1.14)

Some distinctions between faculties are drawn by every human being. If everyone can draw rightly some distinctions, then some other distinctions can be drawn by those who are better at drawing such distinctions.

It's part of the way that Hume distinguishes between the two species of psychology that abstruse psychology doesn't assume the truth of common‐sense psychology, and mental geography is part of abstruse psychology. Still, given that he thinks that common‐sense psychology is mostly right, we should suppose that he expects mental geography to replicate the claims of common‐sense psychology on topics where common sense has an opinion. On such topics, we might think of mental geography as a kind of audit, double checking to make sure that things are as common sense says they are.

Hume uses the faculties of common‐sense psychology as a jumping off point in the first *Enquiry*. The higher‐level mental powers that Hume says everyone distinguishes are will, understanding, and imagination. In the first *Enquiry*, his focus is on understanding and the imagination. He divides the imagination in particular into two parts and then subjects the results to introspection. His method here, I think, is an instance of what Robert Cummins calls “functional analysis,” which “consists in analysing a disposition into a number of less problematic dispositions such that programmed manifestation of these analyzing dispositions amounts to a manifestation of the analysed disposition” (Cummuins 2000, p. 125). That's Hume's method of mental geography.

Hume implicitly divides the imagination between voluntary and associative subcapacities. The voluntary imagination depends on our volitions. In these, an “act of volition… raises a new idea in our imagination” (*EHU* 7.9). The imagination “has unlimited power of mixing, compounding, separating, and dividing these ideas” (*EHU* 5.10). On the other hand, the associative imagination doesn't depend on the will and is governed in part by principles of association. These principles connect ideas “in their appearance to the memory or imagination” so that “they introduce each other with a certain degree of method and regularity” (*EHU* 3.1). Events in a well written composition “must be related to each other in the imagination, and form a kind of *Unity*” (*EHU* 3.6. This division is, I say, the sort of finer distinction that Hume has in mind in his defence of the possibility of mental geography.

Once we divide the faculties into parts, they become possible objects of reflective investigation. Let me begin with Hume's description of the voluntary imagination. The main limitation that Hume offers on the voluntary imagination in Section 3 is the Copy Principle: simple ideas are all copied from similar impressions. In justifying this principle, he argues as follows,

We may prosecute this enquiry to what length we please; where we shall always find, that every idea which we examine is copied from a similar impression. Those who would assert, that this position is not universally true nor without exception, have only one, and that an easy method of refuting it; by producing that idea, which, in their opinion, is not derived from this source.

(*EHU* 2.6)

In every instance we're aware of, simple ideas are copied from similar impressions, and there are no counter examples. This is a simple induction from enumerated instances. Hume also justifies the Copy Principle by appealing to the purported fact that “If it happen, from a defect of the organ, that a man is not susceptible of any species of sensation, we always find, that he is as little susceptible of the correspondent ideas. A blind man can form no notion of colours; a deaf man of sounds.” (*EHU* 2.7). Hume then lists a few other cases of ideational privation (Laplanders don't have ideas of the taste of wine; selfish people can't conceive of generosity.) This is another case of simple induction, with the “singular” exception of the missing shade of blue (*EHU* 2.8).

In Section 7, Hume argues that we don't get an impression of necessity by reflecting upon the voluntary imagination. First, he argues, if we did get such an impression, then we would know how our volitions produce ideas, “But the manner, in which this operation is performed; the power, by which it is produced; is entirely beyond our comprehension” (*EHU* 7.17). Second, the extent of our voluntary control over our perceptions is known “only by experience and observation” (*EHU* 7.18), which wouldn't be the case if volition produced an informative impression of necessity. Third, our control over our ideas varies with respect to our health, the time, and our level of hunger. According to Hume, we can't give “any reason for these variations, except experience” (*EHU* 7.19). He thinks the true story of our impression of necessity is that it is “customary transition of the imagination from one object to its usual attendant” (*EHU* 7.28), and this account applies to both mental and physical causation. We observe constant conjunctions, this sets up an association between perceptions, and the feeling of necessary connection is the established transition between perceptions.

This clarifies what wasn't all that obscure to begin with, namely what Hume means when he says that our knowledge of the scope and limits of the voluntary imagination is a matter of experience. We look inward and see constant conjunctions and correlations between certain volitions and certain perceptions in various circumstances. The principles enunciated in the *Enquiry* apply to perceptions and the relations between them. Hume's arguments for these principles turn on our internal observations of our perceptions and the relations between them, and not on a distinct awareness of the faculties they belong to.

In addition to the voluntary imagination, Hume describes a part of the imagination that's governed by principles of association. He thinks that all his readers will grant that his principles of association “serve to connect ideas” (*EHU* 3.3). That he has given an exhaustive list should be decided by running

over several instances, and examine carefully the principle, which binds the different thoughts to each other, never stopping till we render the principle as general as possible. The more instances we examine, and the more care we employ, the more assurance shall we acquire, that the enumeration, which we form from the whole, is compleat and entire.

(*EHU* 3.3)

Every instance of association of ideas that we examine falls into one of Hume's three kinds, so, very probably, they all do. Again, this is a simple induction from instances.

According to Hume, if a person “has lived so long in the world as to have observed similar objects or events to be constantly conjoined together,” as a consequence “he immediately infers the existence of one object from the appearance of the other” (*EHU* 5.4). Call this pattern the Principle of Customary Inference. Hume does not explicitly rest the Principle of Customary inference on simple induction, but he does say that his description is non‐explanatory. We can call this process, if we like, custom, but “By employing that word, we pretend not to have given the ultimate reason of such a propensity. We only point out a principle of human nature, which is universally acknowledged, and which is well known by its effects” (*EHU* 5.5). This is description, not explanation.

### 2.3 Subfaculties as Processes

In the *Treatise*, Hume implies that when scholastics use the terms ‘faculty’ and ‘occult quality’ they “are wholly insignificant and unintelligible” (*T* 2007, 1.4.3.10; Schafer 2024, pp. 305‐07). I think his point is that faculties aren't explanatory entities that govern the perceptions that fall under them. Hume is assuming some version of Locke's deflationary account of faculties. In the *Essay Concerning Human Understanding*, Locke criticizes those who reify faculties as agents, draws an equivalence between faculties and power, and says that having a power to φ is just a matter of being able to φ: “For nothing can operate, that is not able to operate; and that is not able to operate, that has no *power* to operate” (1690/1975 2.21.20).^4^ Locke continues, “the fault has been, that Faculties have been spoken of, and represented, as so many distinct Agents.” This is, I think, the spirit of Hume's remark: faculties aren't explanatory agents, and they are observable to reflection only when instantiated as perceptions.

Once we understand Hume's method in the *Enquiry* as a matter of breaking faculties into parts and then identifying those parts with processes, we can understand his arguments for one of his central conclusions. The title of Section 5 is “Sceptical Doubts Concerning the Operations of the Understanding,” which is about a faculty. The one proposition he “intended to enforce in the present section” is that “it is not reasoning which engages us to suppose the past resembling the future, and to expect similar effects from causes, which are, to appearance, similar” (*EHU* 4.23), which is about a process. For Hume, argument is the core function of the understanding. If you say that “the understanding of the child” concludes that fire is hot by “any process of argument or ratiocination,” then you have to produce the argument (*EHU* 4.23). It is, Hume tells us at the conclusion of Section 5, more conformable to the ordinary wisdom of nature that causal inference be automatic, rather than the produce of “the laboured deductions of the understanding” (*EHU* 5.22)

In Section 5, the alternative to being determined by the understanding to make the inference is “custom” which is the process that “produces a propensity to renew the same act or operation, without being impelled by any reasoning or process of the understanding” (*EHU* 5.5). A little later, Hume asserts, “the force of custom, carries the imagination to conceive that object, which is usually conjoined to it; and this conception is attended with a feeling or sentiment, different from the loose reveries of the fancy” (*EHU* 5.11). He assumes that custom is an operation of the imagination because he believes that custom arises out of association, and association, he has argued in Section 3, is one of the central functions of the imagination.

Hume's practice of the *Enquiry* is to put effort and argument into describing the processes that govern the mind, but he treats the question of which process belongs to which faculty as obvious and unproblematic. We should think of faculties as the frame through which Hume views the mind, but as a frame that can be removed without affecting his central arguments.

It is possible to combine associationism and faculty psychology, as Hume's example shows. According to Emanuele Levi Mortera (2005, pp. 157–158), British thinkers in the century and a half after Locke's *Essay* are divided between those who think association is the key to understanding human conduct and those who think that it works alongside other principles that are at least as important. Only in the 19th century do we get a sharp division between associationist psychology and faculty psychology (Mortera 2005, p. 159).

In describing the prospects for mental geography in Section 1 of the first *Enquiry*, Hume expresses his optimism regarding mental geography in strong words: “Nor can there remain any suspicion, that this science is uncertain and chimerical; unless we should entertain such a scepticism as is entirely subversive of all speculation, and even action” (*EHU* 1.14). The only way to doubt the possibility of knowledge of mental geography, according to Hume, is to embrace radical Pyrrhonian scepticism.

Hume says that we have to treat mental geography as trustworthy if we aren't going to be Pyrrhonists because he thinks of its generalizations as simple inductions, that is, as arguments of the form: all observed F are G, so, probably, all F are G. In Section 12 of the *Enquiry*, Hume raises the worry about simple induction that “nothing leads us to this inference but custom or a certain instinct of our nature” (*EHU* 12.22). If Pyrrhonists were successful is persuading people to avoid this and similar inferences, “All discourse, all action would immediately cease; and men remain in a total lethargy, till the necessities of nature, unsatisfied, put an end to their miserable existence” (*EHU* 12.23). When Hume says that rejecting the possibility of mental geography is to reject all speculation and action, he means that mental geography is trustworthy if simple induction is trustworthy, and if simple induction isn't trustworthy, then we're in trouble.

It's worth mentioning that in addition to these introspective justifications for the possibility of mental geography, Hume elsewhere suggests history as a source of raw material for the science of man. In the introduction to the *Treatise*, Hume says that the delicacy of our mental operations entails that premeditated reflection will “disturb the operation of my natural principles, as must render it impossible to form any just conclusion from the phænomenon” (*T* 2007, Intro, p. 10). As an alternative, “we must therefore glean up our experiments in this science from a cautious observation of human life, and take them as they appear in the common course of the world, by men's behaviour in company, in affairs, and in their pleasures” (*T* 2007, Intro, p. 10). This will include ordinary observations in ordinary life, but also history. According to Hume, the “chief use” of history “is only to discover the constant and universal principles of human nature, by shewing men in all varieties of circumstances and situations, and furnishing us with materials, from which we may form our observations, and become acquainted with the regular springs of human action and behaviour” (*EHU* 8.7). Historical records, he tells us, “are so many collections of experiments, by which the politician or moral philosopher fixes the principles of his science” (*EHU* 8.7).^5^

According to Karl Schafer (2024, p. 305), the fact that Hume is always ready to assign mental operations to mental faculties indicates that we should think of “mental faculties as forming something close to explanatory bedrock of his new ‘science of man’.” Hume doesn't seem to hesitate in his assignments of processes to faculties, but his assignments can be haphazard. In the *Treatise*, there's an opportunistic definition of imagination where he sometimes contrasts it with memory and sometimes with reason, depending on his purpose. When he speaks of the imagination without contrasting it with another faculty, he promises that “context will sufficiently explain the meaning” (*T* 2007, 1.3.9.18n22).^6^

Something similar happens in the first *Enquiry*. One might think Hume is concerned to show that causal inferences are made by the imagination and not by the understanding. But later in the *Enquiry*, Hume seems willing to attribute causal inferences to the understanding. Observed moral and natural constant conjunctions have the same “operation on the understanding” (*EHU* 8.19). The principle that all we know of causes is constant conjunction provides “limits to human understanding” (*EHU* 8.22). When someone believes in a miracle, his faith, according to Hume, “subverts all the principles of his understanding, and gives him a determination to believe what is most contrary to custom and experience” (*EHU* 10.41). In these passages it seems as if Hume is assigning causal inference to the understanding. The claim he cares about is that causal inference is done by a process that depends on association. Whether he places the process within the imagination or the understanding depends on his mood and purpose.

According to Demeter (2021, p. 5364) “Instead of *from* faculties, Hume argues *to* them; they are not the beginning but the aim of proper, experimental enquiry that reveals the characteristic of faculties.” Once you've described the processes underlying the sub‐faculties, you can assign them to the general faculties as you please. But the fact that Hume treats the assignment of operations to faculties as part of mental geography implies that he thinks of these assignments as descriptive and not explanatory. Describing faculties is still mental geography. Hume's ultimate ambition is something more.^7^

## 3 Newtonian Psychology

### 3.1 Newton as a Model

According to Hume, we may reasonably hope to do better than mental geography. We may, he argues, hope to find explanatory principles. His model for optimism is Newton's account of the solar system:

Astronomers had long contented themselves with proving, from the phænomena, the true motions, order, and magnitude of the heavenly bodies: Till a philosopher, at last, arose, who seems, from the happiest reasoning, to have also determined the laws and forces, by which the revolutions of the planets are governed and directed.

(*EHU* 1.15)

Before Newton, astronomers merely described the motion of the planets. Newton finds the laws and the fundamental force that explains those descriptive patterns.

Hume's goal is to find explanatory mechanisms and principles that lie beneath the surface, “the secret springs and principles, by which the human mind is actuated in its operations” (*EHU* 1.15). After all, “It is probable, that one operation and principle of the mind depends on another; which, again, may be resolved into one more general and universal” (*EHU* 1.15). Since operations of the mind probably stand in explanatory relations to one another, we can probably discover some of these relations.

Hume's distinction between Newtonian psychology and mental geography is partly an expression of ambition: he wants to explain how he has surpassed Butler and Hutcheson. They described the faculties; he has found the laws and forces that explain the operation of those faculties. More importantly, it's a useful thing in psychology to distinguish between the observed phenomena and explanations of those phenomena.^8^

Newtonian psychology is more ambitious than mental geography and correspondingly, Hume has less confidence in it. According to him, “how far” Newtonian psychology “may possibly be carried, it will be difficult for us, before, or even after, a careful trial, exactly to determine” (*EHU* 1.15). That is, even after we've acquired collected evidence for our hypotheses, it can be hard to know whether we are right. All that Hume wants in mental geography is getting general descriptive principles right. In Newtonian science, as Hume conceives of it, one is looking for explanatory depth, and new epistemic dangers loom here. Positing new laws and forces goes beyond mere description and can go wrong in ways that mere description cannot.

Hume doesn't think that the psychological principles that he describes are exceptionless. As John Bricke emphasizes (1974, p. 409), Hume says in the *Treatise* that we should regard the association of ideas “only as a gentle force, which commonly prevails” (*T* 2007, 1.1.4.1) and which govern only “with a certain degree of method and regularity” (*EHU* 3.1). In the *Treatise*, Hume worries that the spread of vivacity across perceptions associated by resemblance and contiguity “is very feeble and uncertain” (*T* 1.3.9.6).

I hesitate to follow Bricke (409) in calling the relevant laws ‘probabilistic,’ however. Hume thinks that there's always a contrary cause that explains exceptions to general psychological principles. Peasants will observe that sometimes clocks don't work. Artisans know that there's an explanation. If we run an induction over such cases, we'll conclude that there's always a hidden cause for every effect: “From the observation of several parallel instances, philosophers form a maxim, that the connexion between all causes and effects is equally necessary, and that its seeming uncertainty in some instances proceeds from the secret opposition of contrary causes” (*T* 1.3.12.5 = *EHU* 8.13). The same phenomenon occurs in intelligent agents. So, “The philosopher, if he be consistent, must apply the same reasoning to the actions and volitions of intelligent agents,” (*EHU* 8.15) and conclude that the human mind is “subjected to the same laws of necessity” as “the operations of matter” (*EHU* 8.32).^9^

Eric Schliesser and Demeter (2020, §3) rightly observe that in the Appendix to the *Treatise*, Hume offers an operationalist interpretation of what the existence of a vacuum means on “the Newtonian philosophy… rightly understood” (*THN* 1.2.5.26n12), namely, that “bodies are said to be plac'd after such a manner, as to receive bodies betwixt them, without impulsion or penetration” (*THN* 1.2.5.26n12). So, according to Schliesser and Demeter (2020, §3), Hume's discussion of Newton in Section 1 of the *Enquiry* “must be interpreted in light of his deflationary commitments.” If we do that, however, and we deflate Hume's Newtonian ambitions, then his distinction between mental geography and Newtonian psychology wouldn't be interesting and important, and it wouldn't make sense for him to frame his ambitions through it. But the distinction is interesting and important, and Hume does frame his work through it. I would rather say, following James Hill (2012, §§4–5), that Hume's later realism about Newtonian forces is one of the main differences between the *Treatise* and the first *Enquiry*.

Hume gives us a version of the contrast between easy and abstruse philosophy in the Abstract of the *Treatise*. There he writes that, *“*the philosophers of antiquity, who treated of human nature… content themselves with representing the common sense of mankind in the strongest lights, and with the best turn of thought and expression” (Abstract ¶1). The step that they don't take is “following out steadily a chain of propositions, or forming the several truths into a regular science” (Abstract ¶1). This suggests that, at least when he writes the Abstract, Hume thinks of the difference between common‐sense psychology and the science of man as a matter of structure and form. The propositions in a science hang together and a science contains first principles out of which others may be derived. In the Abstract, the reason to hope that psychology might become a science is success in natural philosophy more generally: “'tis at least worth while to try if the science of *man* will not admit of the same accuracy which several parts of natural philosophy are found susceptible of. There seems to be all the reason in the world to imagine that it may be carried to the greatest degree of exactness” (Abstract ¶1).

There's a noteworthy shift in Hume's treatment of Newton between the *Treatise* and the first *Enquiry*. As Schliesser writes,

Newton is never mentioned in the *Treatise*; only in the ‘Appendix’, which Hume wrote after he had published the first two volumes, does he use the phrase, ‘Newtonian philosophy.’ In contrast to *EHU*, which has a Newtonian rhetoric, some explicit mention of Newton, and increasing focus on the status of ‘laws’, the *Treatise* is remarkably unaffected by Newtonian themes, concepts, or methods.

(Schliesser 2009, p. 171)

After the *Treatise*, Hume begins to praise Newton in unrestrained terms, calling him, among other things, the greatest genius who ever lived (*H* 1983, 6.362).

We can see the change of tone by comparing the Introduction of the *Treatise* to Section 1 In the Introduction to the *Treatise*, he claims that the sciences of his day are in a parlous state without making any exception. One doesn't need “profound knowledge to discover the present imperfect condition of the sciences” (*T* 2007, Intro ¶2). “Even the rabble,” who don't participate in the sciences but consider it from the outside, “may judge from the noise and clamour, which they hear, that all goes not well within” (*T* 2007, Intro ¶2). The unsatisfactory state of the sciences might be cured if we developed a science of man: “'Tis impossible to tell what changes and improvements we might make in these sciences were we thoroughly acquainted with the extent and force of human understanding, and cou'd explain the nature of the ideas we employ, and of the operations we perform in our reasonings” (*T* 2007, Intro ¶4).^10^

In the first *Enquiry*, Newton's work in astronomy is treated as qualitatively different from what preceded it. His physics doesn't require improvement. It's rather a model for the science of man of what's possible to achieve in the sciences. Just as Newton went beyond mere description and discovered the “laws and forces” that govern the planets, we might hope to find “secret springs and principles, by which the human mind is actuated in its operations” through similar reasoning (*EHU* 1.15).

My conjecture is that Hume becomes more impressed by Newton's achievements during his visits to Edinburgh in the first half of the 1740 s where Colin McLaurin, the great guardian of Newton's legacy, presides over the Edinburgh Philosophical Society. Schliesser (2009, p. 172) makes the good observation that Hume shifts his tone concerning Newton after Maupertuis's return from his expedition to Lapland that confirmed Newton's prediction about the shape of the earth.

### 3.2 Psychological Laws and Forces

Hume's explanatory account of causal inference has four parts. The first is an analysis of belief as a kind of vivid idea. The second is the doctrine advanced in Section 2 that sensations are more vivid perceptions than ideas. The third is a tacit doctrine that the observation of constant conjunction between two sorts of objects establishes an association between the ideas of those objects. The fourth is a putative law that vivacity spreads from impressions to associated ideas. From these principles, he not only explains causal inference concerning unobserved matters of fact, but also other phenomena in the neighbourhood.

The basics of the account are already in the original presentation of the theory in the *Treatise*. There are two differences worth mentioning.

First, in the main part of the *Treatise*, he asserts that perceptions only vary along a single dimension of vivacity (*T* 2007, 1.3.7.5). He's thinking of vivacity as something like the brightness of a mental image. In the Appendix to the *Treatise*, he realizes that he has oversimplified matters and that a piece of poetry might be more vivid than a belief in one respect and less vivid in another (*T* 2007, App ¶22, *T* 1.3.10.10). As a result, he concludes that belief is an idiosyncratic feeling that comes with its own degrees of vivacity (*T* 2007, App. ¶6).^11^ In the first *Enquiry*, Hume repeats material from the Appendix giving his account of belief as a *sui generis* feeling (*EHU* 5.12 = *T* 1.3.7.7).

The second difference is the explanation of causal inference in the *Enquiry* is much abridged relative to the account in the *Treatise*. In Section 1, Hume says that the abstractedness of hard philosophy may be overcome in part by “avoiding of all unnecessary detail” (*EHU* 1.17). For the most part, his explanation makes sense as it stands in the *Enquiry*, but in the *Treatise* he is explicit is saying that the observation of constant conjunction of events leads to a corresponding association of ideas: “when every individual of any species of objects is found by experience to be constantly united with an individual of another species, the appearance of any new individual of either species naturally conveys the thought to its usual attendant” (*T* 2007, 1.3.6.14).

By way of illustration, Hume appeals to the association between words and ideas: “because such a particular idea is commonly annex'd to such a particular word, nothing is requir'd but the hearing of that word to produce the correspondent idea; and'twill scarce be possible for the mind, by its utmost efforts, to prevent that transition” (*T* 1.3.6.14). The constant conjunction of words and the ideas that they signify sets up an associative connection between the two, so that whenever you hear a word, you form the idea. This association doesn't need reflection on past experience, and the connection usually works without “a moment's delay betwixt the hearing of the one, and the conception of the other” (*T* 1.3.6.14). This doctrine isn't new to Hume; Spinoza argues that we come to associate images with words through a process of constant conjunction (1677/1996 *Ethics* 2p18).^12^ The surprising new doctrine Hume adds to this is that this principle of association is “the very same with that betwixt the idea of cause and effect” and is “an essential part in all our reasonings from that relation” (*T* 1.3.6.15).

Hume's explanation of customary inference in Part 2 of Section 5 is supposed to be an attempt at Newtonian psychology. I say this for three reasons: first, because it includes a statement of a possible psychological law; second, because it's supposed to explain the customary causal inference described in Part 1 of Section 5; and, third, because he describes it as being less certain than the claims in the other parts of the *Enquiry*.

The first reason for thinking that Hume is attempting Newtonian psychology in Part 2 of Section 5 is that he calls a principle a potential law governing the mind. Recall that Hume says that Newton's advancement in astronomy is characterized by his discovery of the laws and forces that govern the motion of the planets. The only place that Hume claims to have possibly discovered a general law governing the operations of the mind is in the Part 2 of Section 5, where he offers us the following principle: “when one of the objects is presented to the senses or memory, the mind is not only carried to the conception of the correlative, but reaches a steadier and stronger conception of it than what otherwise it would have been able to attain.” That is to say, if two sorts of perception are associated in the mind, then, if we have a relatively vivid perception of one, then we will form the relatively vivid perception of the other. In a good and sympathetic discussion of Hume's theory, Markus Wild (2011, p. 76) calls this purported law the “Transference Principle.” He tells us that if this principle can be confirmed not only for the associative relation of cause and effect but also for resemblance and contiguity, then it “may be established as a general law, which takes place in all the operations of the mind” (*EHU* 5.14). This hypothesis isn't a mere generalization that rests on other generalization but an explanatory principle that purports to explain other generalizations describing the mind.

Today psychologists conceptualize a descendent of Hume's conception of the transfer of vivacity as priming. According to Mike Dacey 2019, on modern accounts of priming through association,

Priming effects are generally thought to result from the mechanism of association: when a prime is presented, subcritical levels of activation spread to associated representations, which facilitates their use in subsequent tasks. These networks are thought to be composed of links built by associative learning processes, which simply reflect patterns of co‐occurrence in one's experience.

(2019, pp. 281–282)

To be sure, modern psychologists allow for priming to connect states, capacities, and inclinations that aren't present to consciousness. Still, the central doctrines in Hume's cognitive theory, that there are principles of association, that stimulating one of the associated elements can lead to a heightened response in the other without argument or conscious inference, and that these responses include belief are living parts of modern scientific psychology.

The second reason for thinking that Hume is doing Newtonian psychology rather than mental geography is that he is explaining phenomena and not merely describing them. The phenomenon to be explained is the Principle of Customary Inference, the fact that after someone experiences similar objects or events to be constantly conjoined, the appearance of one leads to the expectation of the other. The explanation runs as follows. Hume assumes that the experience of observing two sorts of objects or event being constantly conjoined leads us to associate the idea of one with the idea of the other. He posits as a putative law that vivacity spreads across associated perceptions. He analyses appearances as impressions, which are relatively vivid perceptions, and he analyses beliefs as relatively vivid ideas. It follows on these assumptions that, after we observe the constant conjunction of two sorts of things, the appearance of one will lead to the expectation of the other.

Other examples in the Second Part are intended to show that vivacity also transfers along other principles of association. When we consider the resembling picture of an absent friend, all the emotions that the friend produces are produced by the image (*EHU* 5.15). If the son of a long‐absent friend is presented to us, our idea of the friend will be revived in more lively colours, and we'll recall “past intimacies and familiarities” (*EHU* 5.19). When we are near home, the feelings we have concerning it affect us more (*EHU* 5.17). The iconography and sensible images of Catholicism produce more faith than intellectual contemplation (*EHU* 5.16). Superstitious people are fond of relics because effects are associated with causes, and the sensations of the relics enliven their conception of the saint (*EHU* 5.18).^13^ In the last two cases, the data is gathered from history and other second‐hand reports. Hume isn't looking inward and finding faith from Catholic relics.

In each of these cases the vivacity of the stimulus is the vivacity of sensation, whatever exactly that amounts to. The transferred vivacity generates belief, including expectation, memory, and more intense feelings, including greater religious devotion.

In the analogy between Humean associationism and Newtonian astronomy what are the forces supposed to be? To some extent, we are spoiled for choice. Hume very often conjoins the terms “force and vivacity” in his discussions of perceptions, as if he treats these as alternate words for the same thing (e.g. *T* 1.1.1.3, 1.3.5.3, 1.3.8.2, *EHU* 2.1, 2.3). To be sure, psychological intensity is different from Newtonian force, but Hume's willingness to use ‘force’ as another word for ‘vivacity’ suggests that he might conceptualize them in similar ways. In addition, Hume says in the *Treatise* that the principles of association provide “a kind of Attraction, which in the mental world will be found to have as extraordinary effects as in the natural, and to shew itself in as many and as various forms” (*T* 2007, 1.1.4.6), which suggests that he thinks of association as being something like gravitational attraction.^14^ Both vivacity and strength of association come in degrees and allow for generalizations that turn on matters of degree.

Hume thinks that we discover these forces through a process of generalization and simplification. On the physical side, in a place where he's describing our inability to find ultimate explanations, he describes the project for finding fundamental causes as “to reduce the principles, productive of natural phænomena, to a greater simplicity, and to resolve the many particular effects into a few general causes, by means of reasonings from analogy, experience, and observation” (*EHU* 4.12). The fundamental causes he lists here are “Elasticity, gravity, cohesion of parts, communication of motion by impulse” and “we may esteem ourselves sufficiently happy, if, by accurate enquiry and reasoning, we can trace up the particular phænomena to, or near to, these general principles” (*EHU* 4.12). This is Hume's account of how we discover basic physical forces.

On the psychological side, in enumerating the principles of association, he writes, “All we can do, in such cases, is to run over several instances, and examine carefully the principle, which binds the different thoughts to each other, never stopping till we render the principle as general as possible” (*EHU* 3.3). In an appended footnote, he gives an illustration:

For instance, Contrast or Contrariety is also a connexion among Ideas: But it may, perhaps, be considered as a mixture of *Causation* and *Resemblance*. Where two objects are contrary, the one destroys the other; that is, is the cause of its annihilation, and the idea of the annihilation of an object, implies the idea of its former existence.

(*EHU* 3.3n6)^15^

In both cases, enquiry proceeds by finding explanatory principles and then reducing the number of basic principles by establishing hierarchical explanatory relations between higher‐level principles and lower‐level ones. The basic explanatory principles are, I think, what he has in mind when he aspires to finding the forces that govern the mind in Section 1 of the *Enquiry*.

The third reason for thinking that Hume is doing Newtonian psychology in Part 2 of Section 5, is that he describes the part as optional and speculative in a way that Part 1 is not. For readers who don't like uncertain speculations, “the remaining part of this section is not calculated for them, and the following enquiries may well be understood, though it be neglected” (*EHU* 5.9). Part 2 is offered for those who “love the abstract sciences, and can be entertained with speculations, which, however accurate, may still retain a degree of doubt and uncertainty” (*EHU* 5.9). Recall that Hume describes mental geography as having a high degree of certitude. The speculative character of his discussion is a sign that we have left mental geography behind, and he is attempting to explain customary inference.

I don't think Hume thinks that every appeal to a law is less certain than mere descriptive generalization. He isn't impugning the certitude of Newton's laws by implying that his own proposed law is conjectural.^16^ In his contrast between miracles and marvels in his argument against believing in miracle stories, he implies that well‐confirmed laws are more certain that mere exceptionless generalizations (*EHU* 10.10‐11). Perhaps Hume thinks that there's more epistemic variance in claims about laws than there is in mere description. A putative law is less certain than an exceptionless generalization when first proposed, but after a suitable level of confirmation becomes more certain. In the present case, he thinks that mental geography has the epistemic security of simple induction, and his conjectural law is less certain than that.

According to Justin Broackes (2002, p. 187), Hume's statement that Part 2 of Section 5 is optional is evidence of a loss of confidence in his theory of belief: “Hume's anxieties seem to have gotten the better of him altogether.” There is something right about this, but I wouldn't personalize the point. Hume thinks that his hypothetical law is less certain than his mental geography, but it's also more ambitious. He makes his speculation optional so that its uncertainty doesn't infect the rest of the work, but he still thinks of it as an attempt to do for the science of mind what Newton did for astronomy.

What, in the end, is the difference between mental geography and a Newtonian psychology? A science of mind, on Hume's picture, would be explanatory and it would appeal to laws and forces. It may start out as conjectural and uncertain, but if confirmed by reason and experience, it may end up as more trustworthy and reliable. His best attempt at a Newtonian science of mind is the account of causal inference that he gives in the Second Part of Section 5 of the first *Enquiry*.

I don't think that the Newtonian ideal of laws and forces isn't part of the story of how Hume came up with his account of causal inference. It rather is Hume's later and considered view of how his explanatory account of causal inference should be defended, advertised, and understood.

## Conflicts of Interest

The author declares no conflicts of interest.

## Data Availability

Data sharing not applicable to this article as no datasets were generated or analysed during the current study.

## Endnotes

1 Buckle (2001) gives a good and accurate paraphrase of this section, pp. 121‐5.

2 Lapointe (1970, pp. 640‐1)

3 Lapointe (1970, p. 643)

4 Cummins (2000, pp. 122‐3) offers a similar analysis of psychological capacities

5 See Demeter (2016, pp. 120‐3)

6 Costelloe surveys several inconsistent attempts by commentators to uniquely characterize the functions of the imagination (2018, pp. 38‐9nn6‐7).

7 Hume's implication that mental geography isn't explanatory doesn't mean that he denies its importance. One might reasonably think that a description of a what our faculties do is part of his justification of the deliverances of that faculty. See, e.g., Schafer 2024, p. 320), Wild (2011, pp. 72‐5), and Qu (2020, pp. 79‐95, 184‐99).

8 Qu (2020, pp. 37‐8) observes that the contrast between mental geography and Newtonian psychology doesn't correspond to anything explicit in the *Treatise*

9 On this argument, see Millican (2010, §7).

10 For one account of the nature of this grounding project, see Boehm (2016).

11 See Jacovides (2024). In the Appendix, Hume is very concerned to defend a one‐perception view that a belief is an idea with a certain character, as opposed to a two‐perception view, where belief is an idea with an annexed impression. In the *Enquiry*, he says things that cut both ways, first that “the difference between *fiction* and *belief* lies in some sentiment or feeling, which is annexed to the latter, not to the former” (*EHU* 5.11), which suggests the two‐perception view, but also “belief is nothing but a more vivid, lively, forcible, firm, steady conception of an object” (*EHU* 5.12), which suggests the one‐perception view. According the Stacy Hansen (1988, 299) and Daniel Flage (1990, 181‐2), in the *Enquiry*, Hume comes to accept the two‐perception view that he had attacked in the Appendix, the view that belief consists in an annexed impression. According to Michael Gorman, Hume's talk in the *Enquiry* of the difference between mere conception and belief consisting in an annexed feeling lends support to Hansen and Flage's interpretation, but this passage is something of a lapse, and “Hume soon returns to discussing belief as if the manner of conception or feeling were internal to the belief‐idea” (Gorman 1993, 94). I think that variations in Hume's expressions shows that he had come to realize that the question didn't matter much.

12 Klever (1990, 93) cites this passage as part of an argument that Spinoza influences Hume.

13 In the original version of Section 3, Hume says that a similar phenomenon can be observed in epic poetry, where one's feelings for one character are transferred to associated characters (*EHU* 3.12), anticipating the putative law in Section 5.

14 Demeter (forthcoming, pp. 7‐8) has denied this, but later in the *Treatise*, Hume asserts that “heat, cold, light, attraction, &c. diminish in proportion to the distance” (*T* 2007, 1.2.5.17). The relevant kind of attraction has to be gravitational, since people at the time wouldn't have opinions about how chemical affinity is related to distance. Also, chemical affinity is an esoteric notion in the first half of the eighteenth century and gravitational attraction is a famous one, so if Hume has the first concept in mind, his readers wouldn't know it.

15 I'm indebted to a referee for the example.

16 Here I'm indebted to Zvi Biener.
